# Association Between Relative Fat Mass and Parkinson’s Disease in American Adults: A Large Cross‐Sectional Study From NHANES

**DOI:** 10.1155/padi/2954515

**Published:** 2026-10-06

**Authors:** Quanhong Chu, Wenfeng Cao

**Affiliations:** ^1^ Department of Neurology, Jiangxi Provincial People’s Hospital, The First Affiliated Hospital of Nanchang Medical College, No, 266 Fenghe North Avenue Honggutan District, Nanchang 330038, Jiangxi, China, jxsrmyy.cn; ^2^ Department of Neurology, Xiangya Hospital, Central South University, Jiangxi (National Regional Center for Neurological Diseases), No, 266 Fenghe North Avenue Honggutan District, Nanchang 330038, Jiangxi, China, csu.edu.cn

**Keywords:** cross-sectional study, NHANES, obesity, Parkinson’s disease, relative fat mass

## Abstract

**Background:**

Numerous studies have sought to investigate the link between obesity and Parkinson’s disease (PD), yielding conflicting results. Relative fat mass (RFM) is a novel obesity indicator, yet its association with PD in American adults remains unexplored. This study was designed to explore the potential relationship between RFM and PD in American adults aged 40 and older.

**Methods:**

In the present study, we ultimately included 26, 029 participants from the NHANES (1999–2018). In order to evaluate the association between RFM and PD in American adults aged 40 and older, we performed multivariate logistic regression analysis, restricted cubic spline analysis, subgroup analysis, and sensitivity analysis. Furthermore, we conducted receiver operating characteristic (ROC) curve analysis to evaluate the predictive performance of RFM for PD.

**Results:**

Of the 26, 029 participants who were included in the study, 245 participants (0.94%) were identified as having PD. After full adjustment for confounding variables, the multivariate models demonstrated a significant positive association between RFM and PD (odds ratio [OR] = 1.40; 95% confidence interval [CI] = 1.11 – 1.77). Specifically, participants in the highest RFM quartile (Q4) had a significantly higher prevalence of PD compared with those in the lowest quartile (Q1) in model II (OR = 2.71, 95% CI: 1.48–4.96). Additionally, subgroup analyses demonstrated that the positive correlation between RFM and PD was significantly more prominent in individuals under the age of 60 (*p* for interaction < 0.05). Finally, ROC curve analysis showed that RFM yielded a higher AUC value of 0.612 in participants under the age of 60.

**Conclusions:**

This study is the first to demonstrate a positive linear association between RFM and PD among American adults aged 40 and older.

## 1. Introduction

Parkinson’s disease (PD), ranking as the second most prevalent neurodegenerative condition globally, is pathologically defined by the progressive loss of dopaminergic neurons and the abnormal accumulation of *α*‐synuclein protein within the substantia nigra pars compacta region of the midbrain, imposing tremendous socioeconomic burdens on affected individuals and societies worldwide. Alarmingly, given the accelerating pace of global population aging, it is projected that the global prevalence of PD will reach 12 to 17 million affected individuals by 2040 [1–6]. Although considerable progress has been achieved in PD investigation, the definitive pathophysiological mechanisms driving PD development have yet to be fully elucidated. This is largely due to the sophisticated crosstalk and synergistic effects between genetic and environmental factors [7–10]. Furthermore, the majority of available therapeutic strategies for PD are predominantly directed toward alleviating clinical symptoms rather than halting or slowing disease progression [11–17]. Hence, it is urgent to develop simple and feasible indicators and to identify targeted preventive measures for high‐risk PD individuals [18–20].

Obesity, affecting approximately 30% of the global population and on the rise, poses a significant threat to human health [21]. The potential correlation between obesity and PD has been investigated in several previous studies, but the available evidence is characterized by significant discrepancies and conflicting results. While some studies suggest that obesity was positively associated with an increased risk of PD incidence [22–25], several recent studies have found that underweight individuals have a higher risk of developing PD [26–29]. In addition, certain studies indicate no substantial correlation between obesity and PD [30, 31]. These conflicting findings across studies can be primarily explained by variations in the characteristics of study participants, the analytical strategies adopted, and the specific anthropometric indicators used to evaluate obesity. Body mass index (BMI) is the most common method for obesity screening and has gained wide application in routine clinical practice. However, conventional metrics such as BMI and waist circumference (WC) may inadequately capture the total body fat distribution and visceral fat accumulation, thereby limiting the accurate evaluation of obesity [31]. In this context, the researchers have proposed a new metric for estimating overall body fat percentage, RFM, which integrates WC and height for enhanced fat mass assessment [32–34]. Recent studies have found that RFM was significantly associated with various diseases such as depression, stroke, and cardiovascular disease [35–37]. Nevertheless, whether RFM is associated with an increased risk of PD in American adults remains poorly understood and requires further investigation.

To fill this important research gap, we conducted a cross‐sectional study using data from the NHANES database to explore the potential association between RFM and PD in American adults aged 40 and older, and to assess its predictive ability for PD. Our findings may contribute to the optimization of PD risk stratification and provide valuable insights for effective preventive measures in high‐risk PD populations.

## 2. Materials and Methods

### 2.1. Study Design and Participants

The present study utilized publicly available data extracted from the NHANES (1999–2018), a nationally representative cross‐sectional investigation that employs a multistage probability sampling design to evaluate the nutritional and health conditions among noninstitutionalized American civilians. The NHANES protocol was approved by the National Center for Health Statistics (NCHS) Ethics Review Board, and written informed consent was provided by all participants prior to data collection. The informed consent process implements a standardized two‐stage tiered informed consent mechanism, including the household interview stage and the mobile examination center stage. In order to ensure the quality of the analyzed data, we have adopted the following exclusion criteria: (1) participants with missing data on RFM (*N* = 16, 843); (2) participants with missing information on drug use (*N* = 619); (3) participants younger than 40 years (*N* = 52, 193); (4) participants with missing data on other covariates (*N* = 5, 632). After applying the aforementioned exclusion criteria, a total of 26, 029 eligible participants were ultimately included in this study. The detailed selection flowchart of the study population is illustrated in Figure 1.

**FIGURE 1:**
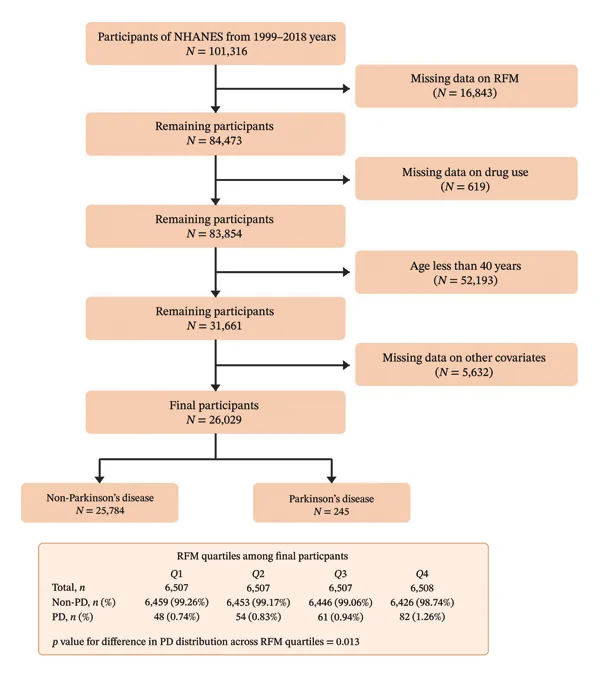
Flowchart of the study population Abbreviations: NHANES: national health and nutrition examination survey; RFM: relative fat mass. A total of 101, 316 participants were initially identified. After excluding participants with missing RFM data (*n* = 16, 843); missing drug use data (*n* = 619); age less than 40 years (*n* = 52, 193); and missing data on other covariates (*n* = 5, 632); 26, 029 participants were included in the final analysis. Among them, 245 participants had Parkinson’s disease and 25, 784 did not. The distribution of participants across RFM quartiles was as follows: *Q*1, *n* = 6, 507 (48 PD cases and 6, 459 noncases); *Q*2, *n* = 6, 507 (54 PD cases and 6, 453 noncases); *Q*3, *n* = 6, 507 (61 PD cases and 6, 446 noncases); and *Q*4, *n* = 6, 508 (82 PD cases and 6, 426 noncases). The difference in PD distribution across RFM quartiles was statistically significant (*p* = 0.013).

### 2.2. Assessment of RFM

In this study, RFM was the primary exposure variable, which was used to evaluate body fat content.

The formula for RFM is as follows:

(1) RFM=64−20×HeightcmWCcm+12×Gender,

where gender is coded as 1 for females and 0 for males [38]. Height and WC were measured by the trained professionals according to the standardized protocols.

### 2.3. Diagnosis of PD

In this study, participants with PD were determined based on the self‐reported use of “anti‐Parkinson medication.” Given the limitations of medication coding in the NHANES dataset, PD was ascertained solely for participants currently receiving treatment with anti‐Parkinson medications. Conversely, participants who did not report undergoing anti‐Parkinson medication were categorized as not having PD. This diagnostic criterion for PD was widely used in previous studies [25, 39].

### 2.4. Covariates

In this study, the covariates include demographic information, socioeconomic information, lifestyle factors, and comorbid conditions. The demographic information encompassed age (years), sex (male or female), and ethnicity, which was classified as Non‐Hispanic White, Non‐Hispanic Black, Mexican American, and other races. The socioeconomic information included education attainment (categorized into three levels: below high school, high school or equivalent, above high school), marital status (defined as married/cohabiting, widowed/divorced/separated, never married), and PIR (poverty income ratio) [divided into three categories: low (PIR < 1.35), medium (1.35 ≤ PIR < 3.0), and high (PIR ≥ 3.0). Additionally, the clinical data, including height, weight, WC, and RFM, were also included in this study.

Information about lifestyle factors included smoking status and drinking status. Smoking status was divided into three categories: never, former, or current smoker. Drinking status was classified into five categories: never, former, mild, moderate, or heavy drinker.

Information regarding comorbid conditions was also taken into account. Stroke was determined based on an individual’s self‐reported previous diagnosis by a physician according to the diagnostic criteria of stroke [40]. Coronary heart disease (CHD) was identified based on self‐reported CHD diagnosed by a physician, adhering to the established diagnostic criteria of CHD [41]. Diabetes (DM) was defined based on any of the following criteria [42]: (1) FPG (fasting plasma glucose) ≥ 126 mg/dL, (2) HbA1c (glycosylated hemoglobin, type A1c) ≥ 6.5%, (3) self‐reported diabetes diagnosed by a physician, and (4) use of antidiabetic medications.

### 2.5. Statistical Analysis

In order to explain the complex multistage probability sampling design of NHANES and ensure the representativeness of the research results, all statistical analyses adopted the appropriate survey weights. According to the analysis guidelines of NHANES, this study reconstructed new 20‐year combined sample weights for the NHANES data spanning from 1999 to 2018.

First, continuous variables were presented as median with interquartile range (IQR), while categorical variables were expressed as frequencies and percentages. All cross‐group comparisons regarding baseline characteristics were evaluated through survey‐weighted linear regression and survey‐weighted Chi‐square tests accordingly.

Second, we utilized three stepwise adjusted logistic regression models to evaluate the potential association between RFM and PD in this study. The crude model was adjusted for no covariates. Model I was adjusted for age and sex. Model II (fully adjusted model) was adjusted for all covariates. Furthermore, in these three models, RFM was divided into quartiles (*Q*1: the lowest value, *Q*4: the highest value), and the Wald test was performed to assess the linear trend of PD risk as the quartiles of RFM increased.

Third, we conducted a restricted cubic spline (RCS) analysis with 4 knots to assess the potential dose–response relationship between RFM and PD in the fully adjusted regression model (Model II). According to Harrell’s guidelines for RCS analysis, this study preset 4 knots, which were placed at the 5th, 35th, 65th, and 95th percentiles of RFM, respectively. Furthermore, in order to enhance the robustness of the results, we performed subgroup analyses to evaluate potential effect modification in the relationship between RFM and PD based on various factors. Meanwhile, we conducted receiver operating characteristic (ROC) curve analysis to assess the predictive ability of RFM for PD. The AUCs of RFM for PD by age group were also compared. The DeLong test was performed to test for significant differences between two age groups.

Finally, to rigorously handle the issue of missing covariates and evaluate the potential selection bias introduced by the main complete case analysis (which excluded individuals with missing data), we performed sensitivity analysis using multivariate imputation by chained equations (MICE). Based on the observed distribution of covariates, we generated four imputed datasets and repeated the multivariate regression analysis considering the survey weights to verify the reliability of our findings.

Statistical analyses in the study were performed utilizing *R* 4.3.1 (R Foundation for Statistical Computing, Vienna, Austria). All *p* values for comparisons were two‐sided, with a significance level set at *p* ≤ 0.05.

## 3. Results

### 3.1. Baseline Characteristics of the Study Participants

The baseline characteristics of the study population stratified by PD are presented in Table 1. Among the 26, 029 participants included in this study, 245 participants (0.94%) had PD. As anticipated, there were statistically significant differences in baseline characteristics between the PD group and the non‐PD group. Specifically, RFM was significantly higher in participants with PD than in those without PD (37.88 ± 8.47 vs. 36.33 ± 8.15). Additionally, the PD group exhibited significantly greater age (64.56 ± 13.30 vs. 59.59 ± 12.45 years), larger WC (104.93 ± 16.09 vs. 100.98 ± 15.18 cm), alongside higher proportions of non‐Hispanic White individuals (68.57% vs. 48.54%) and participants with low PIR (37.55% vs. 28.72%), in comparison to the non‐PD group. In terms of lifestyle factors, the PD group had a higher proportion of former drinkers (35.10% vs. 21.71%), but a lower proportion of never drinkers (13.47% vs. 14.49%), mild drinkers (33.47% vs. 36.55%), moderate drinkers (7.76% vs. 13.33%), and heavy drinkers (10.20% vs. 13.92%) relative to the non‐PD group. Regarding the comorbid conditions, the PD group had a higher prevalence of comorbidities in contrast to the non‐PD group (Stroke: 9.39% vs. 4.85%; CHD: 10.20% vs. 6.08%; DM: 31.02% vs. 23.34%).

**TABLE 1:** Baseline characteristics of participants stratified by presence of Parkinson’s disease.

	Non‐PD	PD	*p* value
Participants	25,784	245	
Age, years	59.59 ± 12.45	64.56 ± 13.30	< 0.001
WC, cm	100.98 ± 15.18	104.93 ± 16.09	< 0.001
Height, cm	166.80 ± 10.10	166.44 ± 10.03	0.581
BMI, kg/m2	29.25 ± 6.41	30.00 ± 6.67	0.070
RFM	36.33 ± 8.15	37.88 ± 8.47	0.003
Sex			0.464
Female	12,970 (50.30%)	129 (52.65%)	
Male	12,814 (49.70%)	116 (47.35%)	
Ethnicity			< 0.001
Non‐Hispanic White	12,515 (48.54%)	168 (68.57%)	
Non‐Hispanic Black	5265 (20.42%)	30 (12.24%)	
Mexican American	4098 (15.89%)	23 (9.39%)	
Other races	3906 (15.15%)	24 (9.80%)	
Education			0.888
Below high school	7170 (27.81%)	71 (28.98%)	
High school or equivalent	5956 (23.10%)	54 (22.04%)	
Above high school	12,658 (49.09%)	120 (48.98%)	
Marital status			0.073
Married/cohabiting	16,388 (63.56%)	146 (59.59%)	
Widowed/divorced/separated	7519 (29.16%)	72 (29.39%)	
Never married	1877 (7.28%)	27 (11.02%)	
PIR			0.002
Low	7405 (28.72%)	92 (37.55%)	
Medium	7837 (30.39%)	77 (31.43%)	
High	10,542 (40.89%)	76 (31.02%)	
Smoking status			0.674
Never	12,853 (49.85%)	124 (50.61%)	
Former	8081 (31.34%)	71 (28.98%)	
Now	4850 (18.81%)	50 (20.41%)	
Drinking status			< 0.001
Never	3735 (14.49%)	33 (13.47%)	
Former	5601 (21.71%)	86 (35.10%)	
Mild	9424 (36.55%)	82 (33.47%)	
Moderate	3436 (13.33%)	19 (7.76%)	
Heavy	3588 (13.92%)	25 (10.20%)	
Stroke			0.001
No	24,534 (95.15%)	222 (90.61%)	
Yes	1250 (4.85%)	23 (9.39%)	
CHD			0.007
No	24,216 (93.92%)	220 (89.80%)	
Yes	1568 (6.08%)	25 (10.20%)	
DM			0.005
No	19,767 (76.66%)	169 (68.98%)	
Yes	6017 (23.34%)	76 (31.02%)	

Abbreviations: BMI, body mass index; CHD, coronary heart disease; DM, diabetes mellitus; PD, Parkinson’s disease; PIR, poverty income ratio; RFM, relative fat mass; WC, waist circumference.

### 3.2. Association Between RFM and PD

The logistic regression results for the association between RFM and PD in three different models are presented in Table 2. In the crude Model (unadjusted), each 1‐SD increase in RFM was significantly associated with a higher prevalence of PD (OR: 1.21, 95% CI: 1.07–1.37), which remained significant after adjusting for age and sex in Model I (OR: 1.49, 95% CI: 1.19–1.86) and after adjusting for all the covariates in Model II (OR: 1.40, 95% CI: 1.11–1.77). Furthermore, stratification of the RFM index into four quartiles was conducted for further analysis. The highest quartile (Q4) of RFM was significantly associated with a higher prevalence of PD relative to the lowest quartile (Q1) in these three models (Model II: OR = 2.71, 95% CI: 1.48–4.96), with all *p* trend < 0.01 Table 2. The dose–response relationships between RFM and PD using RCS analysis in Model II (fully adjusted model) are illustrated in Figure 2. The RCS analysis revealed a significant positive linear relationship between RFM and PD (*p* for nonlinearity: 0.275, *p* for overall: 0.012). Figure 2 also shows the dose–response relationship between RFM and PD by age group. As shown in Figure 2, the RCS analysis also demonstrated a significant positive linear relationship between RFM and PD among different age groups (age < 60 years group: *p* for nonlinearity: 0.835; age ≥ 60 years group: *p* for nonlinearity: 0.189). Notably, in contrast to participants aged 60 and older, the positive association between RFM and PD was more pronounced among participants under the age of 60.

**TABLE 2:** Logistic regression analyses for the association between RFM and the Parkinson’s disease in different models.

Exposure	Or (95%CI)		
	Crude model	Model I	Model II
RFM (per SD)	1.21 (1.07, 1.37)	1.49 (1.19, 1.86)	1.40 (1.11, 1.77)
RFM (quartiles)			
Q1 (7.98–29.96)	1.0 (Ref)	1.0 (Ref)	1.0 (Ref)
Q2 (29.96–35.64)	1.13 (0.76, 1.66)	1.11 (0.75, 1.65)	1.06 (0.71, 1.58)
Q3 (35.64–43.34)	1.27 (0.87, 1.86)	2.01 (1.22, 3.31)	1.81 (1.10, 2.99)
Q4 (43.34–57.29)	1.72 (1.20, 2.46)	3.14 (1.74, 5.64)	2.71 (1.48, 4.96)
*p* trend	0.0018	0.0002	0.0017

*Note:* Crude model adjusted for: None. Model I adjusted for: age and sex. Model II adjusted for: age, sex, ethnicity, education, marital status, PIR, drinking status, smoking status, CHD history, stroke history, and DM history.

Abbreviations: CI, confidence interval; OR, odds ratios; RFM, relative fat mass; SD, standard deviation.

**FIGURE 2:**
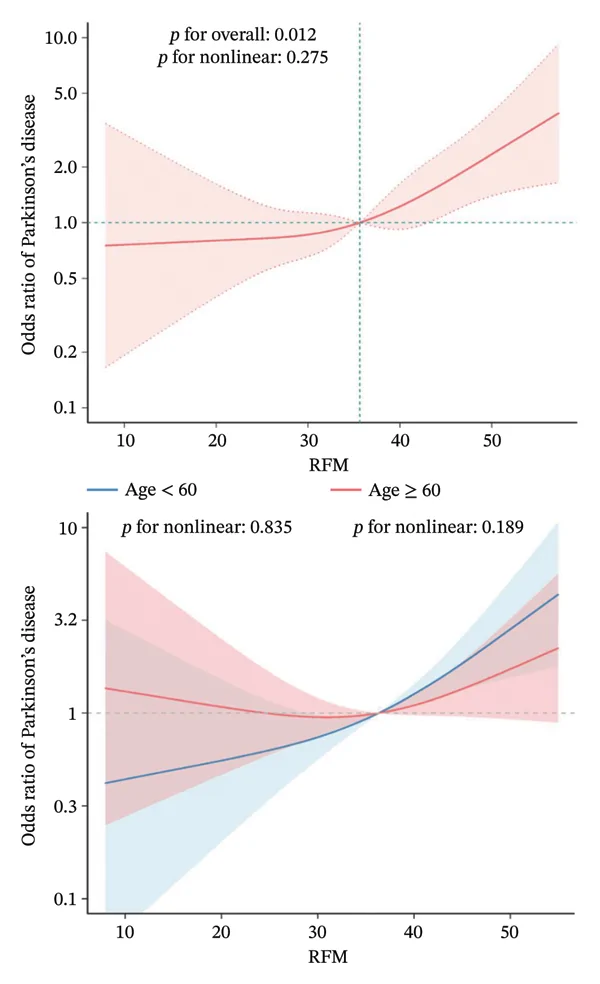
Dose–response relationship between RFM and PD in the entire population and by age group. Abbreviations: PD: Parkinson’s disease; RFM: relative fat mass. The solid red line represents a smooth curve fit between the variables. Blue bars represent 95% confidence intervals for the fit results.

### 3.3. Subgroup Analyses

The stratified relationships between RFM and PD across different subgroup populations are summarized in Figure 3. Notably, there was a significant interaction in the age subgroups (*p* for interaction = 0.02). The association between RFM and PD was more pronounced in individuals under the age of 60 (OR, 1.62; 95% CI, 1.24–2.10). Additionally, the results revealed consistent positive associations between RFM and PD across most subgroups (*p*‐interaction > 0.05).

**FIGURE 3:**
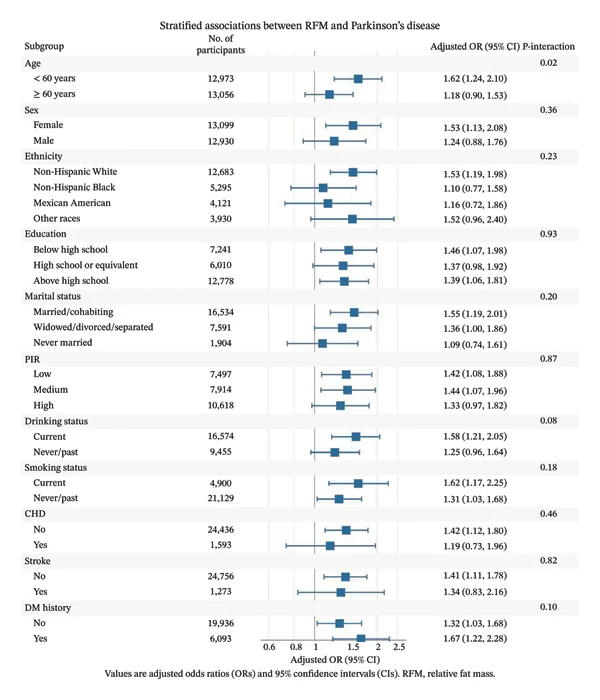
Stratified associations between RFM and the Parkinson’s disease by age, sex, ethnicity, education, marital status, PIR, drinking status, smoking status, CHD, stroke and DM history. Abbreviations: PD: Parkinson’s disease; RFM: relative fat mass; PIR: poverty income ratio; CHD: coronary heart disease; DM: diabetes mellitus; OR: odds ratios; CI: confidence interval.

### 3.4. ROC Analysis

The ROC analyses of RFM diagnosis of PD by age group are shown in Table 3 and Figure 4. Notably, RFM exhibited a superior AUC value of 0.612 in individuals under the age of 60 compared to individuals aged 60 and older (AUC = 0.510). These results suggest that RFM has a statistically significant but relatively low discrimination ability for PD.

**TABLE 3:** ROC analysis of RFM diagnosis of PD by age group.

Test	AUC (95%CI)	Best threshold	Specificity	Sensitivity
RFM				
AGE < 60 years	0.612 (0.553, 0.672)	45.6393	0.8511	0.3505
AGE ≥ 60 years	0.510 (0.462, 0.559)	46.3516	0.8650	0.1959

Abbreviations: AUC, area under curve; CI, confidence interval; PD, Parkinson’s disease; RFM, relative fat mass; ROC: receiver operating characteristic.

**FIGURE 4:**
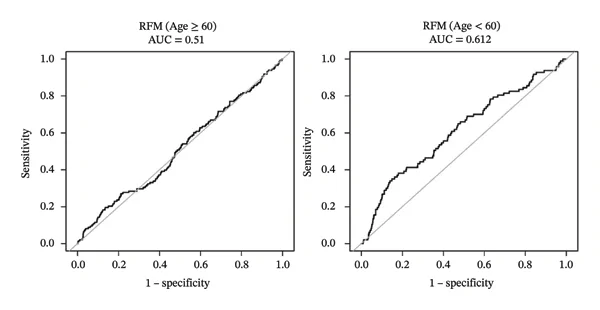
ROC analysis of RFM diagnosis of PD by age group. Abbreviations: ROC: receiver operating characteristic; AUC: area under curve; PD: Parkinson’s disease; RFM: relative fat mass.

### 3.5. Sensitivity Analysis

The results of the sensitivity analysis for the correlation between RFM and PD are presented in Table 4. In all the sensitivity analyses, the positive correlation between RFM and PD remained consistent and was statistically significant. Specifically, when RFM was analyzed as a continuous variable and in quartiles, respectively, we found that there were significant positive associations between RFM and PD across all models. The results of the sensitivity analyses indicate that the positive associations between RFM and PD remain consistent across different statistical analysis methods, thereby enhancing the robustness of our findings.

**TABLE 4:** Sensitivity analysis of the association between RFM and PD.

	Or (95% CI)			
	Sensitivity‐1	Sensitivity‐2	Sensitivity‐3	Sensitivity‐4
RFM (per SD)	1.41 (1.12, 1.79)	1.40 (1.11, 1.77)	1.42 (1.14, 1.81)	1.40 (1.11, 1.78)
RFM (quartiles)				
Q1	1.0 (Ref)	1.0 (Ref)	1.0 (Ref)	1.0 (Ref)
Q2	1.08 (0.74, 1.60)	1.06 (0.71, 1.58)	1.09 (0.76, 1.65)	1.06 (0.71, 1.59)
Q3	1.83 (1.11, 3.01)	1.81 (1.10, 2.99)	1.86 (1.14, 3.13)	1.81 (1.10, 2.99)
Q4	2.72 (1.49, 4.99)	2.71 (1.48, 4.96)	2.74 (1.51, 5.02)	2.71 (1.48, 4.97)
*p* trend	< 0.01	< 0.01	< 0.01	< 0.01

*Note:* (1) Sensitivity‐1 to Sensitivity‐4 were repeated analyses using data after multiple imputations of missing covariates.

## 4. Discussion

Using publicly available NHANES datasets for analysis, this large‐scale cross‐sectional investigation was the first to demonstrate that elevated RFM levels were significantly associated with a higher prevalence of PD in American adults aged 40 and older. Consistent with this finding, RCS regression analyses further confirmed a positive linear relationship between RFM and PD prevalence. Additionally, through stratified analyses, we found the association between RFM and PD was more pronounced among individuals under the age of 60. In summary, these findings have important implications for clinicians, suggesting that these findings highlight the relevance of central obesity is relevant to PD pathogenesis. The present findings may provide potential value for refining PD risk stratification strategies and advancing targeted preventive interventions among high‐risk PD populations.

Obesity, a serious social problem, has already imposed significant economic and social burdens on society. The proinflammatory characteristics of obesity may heighten susceptibility to neurotoxins and reduce dopamine receptor availability, potentially increasing the risk of developing degenerative diseases including PD [31]. The escalating obesity rates have spurred considerable interest in the link between PD and obesity in recent years [27, 28, 31]. However, several previous studies investigating this association have yielded inconsistent and contradictory findings. While some studies revealed a positive relationship between obesity and PD risk [22–24, 43], other studies found that obesity was significantly associated with a decreased risk of PD [26–29]. Surprisingly, certain prospective studies and meta‐analyses showed a null association between obesity and PD risk [30, 31]. These discrepant findings across investigations may stem predominantly from differences in the study populations, statistical analysis methods, and the choice of anthropometric parameters for obesity assessment. Although BMI functions as the primary screening tool for obesity in clinical settings, it is important to recognize that traditional anthropometric metrics such as BMI and WC fail to precisely reflect the distribution of body fat and visceral fat accumulation [31, 32, 44]. Moreover, these indicators cannot accurately evaluate central obesity, which is closely related to metabolic dysfunction and inflammation. Consequently, this can partially explain the reasons for the inconsistent results. Notably, a large‐scale Asian cohort study revealed that higher WC and abdominal obesity were linked to elevated PD risk [45]. Recently, novel screening tools have emerged to better assess the characteristics associated with central obesity comprehensively. For instance, RFM offers a more precise evaluation of body fat percentage and distribution in contrast to BMI and WC [32]. Accumulating evidence from previous investigations has demonstrated that RFM exhibited a significant positive correlation with a spectrum of diseases, including depression, stroke, and cardiovascular disease [35–37]. Nonetheless, the potential association between RFM and PD in American adults remains poorly understood and requires further in‐depth exploration.

In this study, after adjusting for various confounding variables, the results revealed a positive linear relationship between RFM and PD prevalence in American adults aged 40 and older. These findings align with previous research by R.D. Abbott, who observed a positive association between increased triceps skinfold thickness in midlife and PD risk [46]. Furthermore, a large‐scale Asian cohort study found that higher WC and abdominal obesity were significantly linked to an increased risk of PD. Even in individuals without obesity, abdominal obesity was positively associated with an elevated risk of PD [45]. Three cohort studies further supported these findings by demonstrating a significant positive relationship between WC and PD [31, 47, 48]. Conversely, several population‐based investigations have reported a null relationship between WC and PD [49, 50]. Current research evidence in the field of PD genetics may partly explain these conflicting results, suggesting that ethnicity‐specific mutations may play varying roles in the development of PD. Additionally, the distribution of visceral fat may have a different impact on PD risk based on ethnicity and genetic factors [51, 52].

The precise pathophysiological mechanisms linking central obesity to PD remain poorly elucidated, even with the cumulative evidence generated by our investigation and previous research. Insulin resistance (IR) might be regarded as an important metabolic link between central obesity and PD. Insulin signaling impairment plays a key role in promoting the accumulation of intracellular protein aggregates, particularly alpha‐synuclein protein in the substantia nigra, contributing to the pathogenesis of PD [53, 54]. This study is the first to explore the association between RFM and PD in American adults aged 40 and older. Moreover, RFM is a noninvasive and simple indicator that is very easy to obtain in routine clinical practice. From a clinical perspective, RFM holds significant clinical importance for early PD risk stratification and the implementation of targeted prevention measures. RFM could provide valuable insights into the early risk stratification for individuals at high risk of PD.

In subgroup analyses, RFM exhibited a markedly more pronounced positive association with PD in the subgroup of participants under the age of 60. This finding suggests that age might be an important effect modifier for the association between RFM and PD. The possible reasons for this difference might be as follows: First, previous studies have reported that aging is the predominant risk factor underlying the pathogenesis of PD [55, 56]. However, growing evidence indicates that individuals with early‐onset PD display a distinct metabolic signature characterized by enhanced mitochondrial impairment, IR, and altered lipid metabolism that directly impair dopaminergic neuronal survival, alongside a lean phenotype with preserved muscle but reduced fat mass [57]. Relative preservation of skeletal muscle mass in early disease stages makes RFM an excellent sensitive indicator of metabolic dysfunction. Therefore, in younger individuals, RFM can better capture adiposity‐related metabolic stress that may contribute to the initiation of neurodegeneration, whereas in older individuals, age‐related muscle loss and general wasting dilute the specific association between RFM and PD. Our observation that RFM was more strongly associated with PD in younger individuals suggests that adiposity‐related metabolic disturbances may play a more prominent etiological role in the early‐onset spectrum of PD, whereas the etiology of late‐onset PD is more heavily influenced by aging, neurodegeneration, and cumulative environmental exposures. The stronger association between RFM and PD in participants under the age of 60 may reflect distinct metabolic and body composition phenotypes in early‐onset PD. Thus, the stronger association between RFM and PD in participants under the age of 60 is biologically consistent with the distinct metabolic vulnerability of the early‐onset PD phenotype. Second, compared with the elderly population, the metabolic inflammation in the middle‐aged and young adults is more severe [58]. In addition, growing evidence confirms that obesity exerts a profound proinflammatory effect; consequently, the persistent inflammatory milieu induced by obesity directly contributes to the pathogenesis of IR, potentially increasing the risk of developing degenerative diseases including PD [28, 59]. Furthermore, some previous studies have revealed a robust association between obesity‐induced systemic IR and PD, mediated primarily by brain insulin dysregulation [60]. RFM, as an indicator of visceral obesity, is positively associated with IR. Hence, individuals in advanced age groups appear to partially offset some of the detrimental metabolic risk associated with RFM. Third, selection bias or survival bias cannot be excluded given the cross‐sectional design, and this finding should be considered exploratory. This finding holds significant clinical and public health implications. This finding highlights that greater clinical attention should be directed toward individuals under the age of 60 when performing risk stratification in populations at high risk for PD. Accordingly, future PD prevention strategies should prioritize the implementation of RFM‐level screening and management in this age group.

The potential pathophysiological mechanisms responsible for the observed association between RFM and PD may be summarized as follows. First, elevated circulating free fatty acids, proinflammatory cytokines, and ectopic adipose tissue accumulation collectively trigger a state of chronic low‐grade systemic inflammation, which in turn drives the development of IR and accelerates the progression of neurodegenerative processes [61]. There is evidence that IR develops not only in peripheral tissues such as skeletal muscle, liver, and adipose tissue but also in the neuronal and glial compartments of the central nervous system (CNS) [62]. Second, obesity‐induced low‐grade peripheral inflammation can initiate hypothalamic local inflammatory responses, which disrupt synaptic plasticity and accelerate neurodegenerative cascades [63]. Third, obesity‐associated oxidative stress promotes excessive generation of reactive oxygen species (ROS) that specifically target mature oligodendrocytes, resulting in myelin loss in the CNS. Consequently, obesity‐related ROS generation impairs cerebrovascular endothelial function, which compromises white matter structural integrity and accelerates neurodegenerative processes [64, 65]. Lastly, obesity and its metabolic alterations have been associated with compromised brain health and the development of neurodegenerative disease, including PD [66]. In summary, the pathophysiological pathways underlying the association between RFM and PD predominantly involve IR and its interconnected pathogenic mediators, including chronic inflammation and oxidative stress.

This study exhibits some key advantages. First, it is the first study to evaluate the correlation between RFM and PD in American adults aged 40 years and above. Second, it capitalized on a substantial sample size from the NHANES database, which bolstered the credibility of findings. Third, in order to strengthen the reliability and robustness of our study results, we performed comprehensive subgroup analyses and rigorous sensitivity analyses that accounted for a broad spectrum of potential confounding factors.

Despite its aforementioned strengths, several limitations of this study must be acknowledged. First, the cross‐sectional design precludes determination of temporal sequence between RFM and PD. Therefore, the observed differences in fat distribution and metabolic parameters may reflect disease‐related changes rather than antecedent exposure, and causal inferences cannot be established. Well‐designed prospective cohort investigations are warranted to definitively establish the causal association between RFM and PD. Second, potential residual confounding may exist because detailed dietary intake variables (e.g., total energy, total fat, saturated fat, and animal fat intake) were not included in the adjustment model due to high missing rates in the NHANES. Therefore, unmeasured dietary factors might influence the observed association between RFM and PD. In future research, we should take into account the detailed dietary intake variables in our statistical analysis models. Third, the diagnosis of PD was based solely on the use of antiparkinsonian medications, which may have introduced potential misclassification. Therefore, future studies should attempt to adopt a composite diagnostic strategy that integrates clinical interviews, standardized neurological examinations, and validated biomarker assays, thereby improving the precision of PD classification. Lastly, these conclusions are limited to American adults aged 40 years and older, and further research is required to confirm whether these results extend to other racial and ethnic groups.

## 5. Conclusions

In conclusion, our study is the first to reveal a positive linear relationship between RFM and PD in American adults aged 40 and older. These results highlight the potential value of central obesity in the pathogenesis of PD. In the future, RFM may hold significant promise for the early preliminary identification and risk stratification of PD in high‐risk populations. Meanwhile, we need more large prospective studies to validate the findings in clinical practice.

## Author Contributions

Wenfeng Cao was responsible for conceptualization, methodology, supervision, and project administration, and Quanhong Chu wrote the main manuscript text, developed the software, conducted formal analysis, validation, and curated the data.

## Funding

This study was supported by National Health Commission Medical and Health Science and Technology Development Center “Research on Prevention and Treatment Techniques of Stroke” (No. WKZX2023CZ0124); Science and Technology Project of the Health Commission of Jiangxi Province (No. 202610164); Science and Technology Project of Nanchang Medical College (No. NYXJ‐2025‐031).

## Disclosure

All authors read and approved the final manuscript.

## Ethics Statement

All participants provided written informed consent approved by the NCHS Ethics Review Board (https://www.cdc.gov/nchs/nhanes/index.html). The secondary analysis was exempt from additional Ethics Review Board approval.

## Consent

Please see the Ethics Statement.

## Conflicts of Interest

The authors declare no conflicts of interest.

## Data Availability

The data are available at https://www.cdc.gov/nchs/nhanes/index.html.
